# The impact of technology on impaired awareness of hypoglycaemia in type 1 diabetes

**DOI:** 10.1177/20420188251346260

**Published:** 2025-06-12

**Authors:** Simon A. Berry, Iona Goodman, Simon Heller, Ahmed Iqbal

**Affiliations:** Division of Clinical Medicine, School of Medicine and Population Health, University of Sheffield, Sheffield, UK; Division of Clinical Medicine, School of Medicine and Population Health, University of Sheffield, Sheffield, UK; Division of Clinical Medicine, School of Medicine and Population Health, University of Sheffield, Sheffield, UK; Division of Clinical Medicine, School of Medicine and Population Health, The Medical School, University of Sheffield, Beech Hill Road, Sheffield S10 2RX, UK

**Keywords:** automated insulin delivery, continuous glucose monitoring, hybrid closed loop, impaired awareness of hypoglycaemia (IAH), severe hypoglycaemia

## Abstract

Iatrogenic hypoglycaemia remains a major barrier to optimal glycaemic control required to prevent long-term complications in people with type 1 diabetes (pwT1D). Hypoglycaemia is the consequence of the interaction between absolute or relative insulin excess from treatment and compromised physiological defences against falling plasma glucose. With a longer duration of diabetes and repeated exposure to hypoglycaemia, pwT1D can develop impaired awareness of hypoglycaemia (IAH). IAH increases the risk of severe hypoglycaemia six-fold, causing significant morbidity, and, if left untreated, death. Over the last few decades, a stepwise change in diabetes management has been the introduction and widespread uptake of novel technologies, including continuous glucose monitoring (CGM) and automated insulin delivery (AID) systems. These technologies aim to improve glycaemic control whilst minimising hypoglycaemia. Alarms and safety functions, such as suspension of insulin delivery, can help to reduce the hypoglycaemia burden. This review examines the role of continuous glucose monitors and AID systems in managing IAH, exploring evidence for their impact on symptomatic awareness and identifying areas for future research. In conclusion, there is strong evidence that CGM and AID systems improve glycaemic control and reduce the hypoglycaemia burden. However, despite the use of these technologies, severe hypoglycaemic episodes are not entirely eliminated, and it remains unclear whether their implementation restores the physiological symptoms and counter-regulatory response to hypoglycaemia.

## Plain language summary

Staying safe from low blood sugar—the role of technology

Type 1 diabetes occurs when the immune system damages cells in the pancreas that make and release insulin, a hormone needed to control sugar (glucose) levels in the blood. For people with type 1 diabetes, insulin treatment is essential and must be given by injection under the skin. Injections do not control glucose levels automatically and it is hard to match insulin doses to food and activity, resulting in both high and low blood sugar levels (hypoglycaemia). Avoiding high blood sugar is important to prevent long-term complications such as eye disease, whereas low blood sugar is dangerous as the brain needs a continuous sugar supply to function. If this supply is interrupted, it can stop the brain working normally and may lead to confusion, unconsciousness and, very rarely, death. When blood sugar drops, the body releases chemical messengers that can raise glucose levels and cause symptoms warning people their glucose is falling. In long-standing type 1 diabetes, some people lose early symptoms of low blood sugar, leading to a condition called impaired awareness of hypoglycaemia. This makes them more likely to develop severely low glucose levels. New technologies like continuous glucose monitors (CGMs) and automatic insulin delivery systems (AID, sometimes known as the artificial pancreas) have revolutionised diabetes management. CGM can track glucose levels continuously and warn people when levels are falling, while AID systems automatically adjust insulin delivery, aiming to keep blood sugar within a safe range. This review examines the evidence behind CGM and AID systems in treating people with impaired awareness of hypoglycaemia. While these technologies improve blood sugar control and reduce hypoglycaemic events, they do not completely eliminate them. Importantly, it remains unclear whether these devices help people regain the ability to recognise early warning signs of low blood sugar.

## Introduction

**Figure 1.**
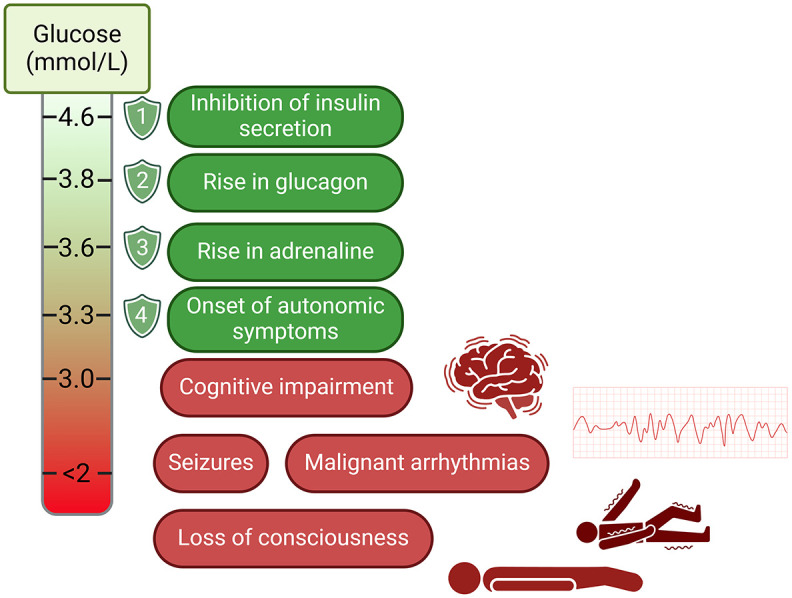
Illustration of the hierarchy of glycaemic thresholds for counter-regulation and complications in hypoglycaemia.^15,16^ Source: Created with biorender.com.

Iatrogenic hypoglycaemia remains a major barrier to optimising glucose levels in people with type 1 diabetes (pwT1D),^1^ which is key to preventing long-term complications.^2^ Hypoglycaemia is the consequence of the interaction between relative insulin excess from treatment and compromised physiological defences against falling plasma glucose.^1^ With a longer duration of diabetes and with increased exposure to hypoglycaemia, pwT1D can develop impaired awareness of hypoglycaemia (IAH). IAH is a clinical syndrome characterised by a diminished ability to perceive the symptoms of hypoglycaemia before the onset of cognitive dysfunction. IAH exists on a clinical spectrum, with complete unawareness being rare. The key pathophysiological abnormality is a blunted counter-regulatory response to hypoglycaemia.^3^ The prevalence of IAH has been estimated to be 27% in pwT1D and 14% in people with type 2 diabetes (T2D),^4^ although there is some evidence that the prevalence is decreasing.^5,6^ Under physiological conditions, the first line of defence against low blood glucose is inhibition of insulin secretion as glucose levels fall, but this is absent in type 1 diabetes (T1D) as insulin needs to be given exogenously in the absence of β-cell secretion (Figure 1). In the absence of the ability to ‘switch off’ insulin, pwT1D rely on glucagon secretion and sympathoadrenal activation to both raise blood glucose and initiate hypoglycaemic symptoms. However, early in the course of T1D, there is loss of the glucagon response to hypoglycaemia, despite preserved secretion to other stimulants such as amino acids.^7^ The third line of defence is catecholamine counter-regulation, which drives adrenergic symptoms and gluconeogenesis. As the hypoglycaemia burden increases, repeated episodes of hypoglycaemia lead to a blunted adrenergic response to further episodes of hypoglycaemia.^8^ This culminates in attenuated adrenaline secretion, lower endogenous glucose production and a reduction in adrenergic symptoms such as sweating, tingling and shakiness during hypoglycaemia.^1^ This is reflected in a lowering of the glycaemic threshold marking sympathoadrenal activation, to below the glucose level at which neuroglycopenia develops. The consequences of neuroglycopenia, including cognitive dysfunction, may prevent self-treatment of a hypoglycaemic episode, resulting in reliance on third-party assistance for treatment. As a result, IAH is associated with a six-fold increased risk in episodes of severe hypoglycaemia (SH), compared to people with normal awareness of hypoglycaemia (NAH).^9^ SH is defined as hypoglycaemia leading to severe cognitive impairment thus requiring external assistance for recovery.^10^ If left untreated, an episode of SH can be fatal, with an estimated 8% of deaths in pwT1D aged under 55 due to SH.^11^ Psychological consequences in those suffering from frequent hypoglycaemia include higher rates of anxiety and depression, and a reduced quality of life.^12^ Furthermore, there are both direct economic costs from treatment expenses and indirect economic costs as a result of reduced productivity.^13,14^

In the last few decades, new technologies have been developed for diabetes care. Continuous glucose monitoring (CGM) sensors measure interstitial glucose.^17^ Automated insulin delivery (AID) systems vary in their capabilities but broadly consist of three features: an insulin pump, a CGM and an algorithm which determines the rate of insulin infusion.^18^ Both these treatments aim to improve glycaemic control whilst reducing the overall burden of hypoglycaemia and, importantly, the frequency of SH. In this review, we will first examine the impact of CGM and AID in the management of IAH, and then address the question of whether these technologies can support the restoration of awareness of hypoglycaemia.

## Assessing treatment response in IAH

Clinically, IAH is diagnosed by the use of well-established and validated questionnaires developed in the 1990s, including the Gold score and the Clarke questionnaire.^19,20^ The Gold score asks a single question ‘Do you know when hypos are commencing?’, answered on a scale from 1 (always) to 7 (never).^19^ A score of ⩾4 is deemed diagnostic of IAH, and a score of ⩽2 is designated as normal awareness. The Clarke questionnaire asks seven questions, including markers of both hypoglycaemia symptoms and frequency.^20^ A Clarke score of ⩾4 is deemed diagnostic of IAH. Given the inclusion of frequency of SH in the Clarke questionnaire, it can show improvement in IAH when interventions reduce the frequency of SH, even if symptomatic awareness has not improved.^21^ In research studies, the symptom and hormonal counter-regulatory responses to hypoglycaemia can be assessed in hyperinsulinaemic–hypoglycaemic clamp experiments, in which precise glucose targets can be achieved via fixed-rate insulin infusions and variable-rate glucose infusions.^22^ This facilitates comparison between participants and within participants before and after an intervention, in a controlled setting. Assessing symptomatic awareness through hyperinsulinaemic–hypoglycaemic clamp studies is a more objective approach, although clearly more labour-intensive and impractical when assessing large numbers. However, the hypoglycaemia challenge in clamps is an experimental model that may not accurately reflect the depth or duration of hypoglycaemia experienced in real-world settings, and it does not account for everyday distractions that might attenuate symptom perception.^15^ Both questionnaires and clamp studies are useful in research to assess reversibility, despite having these limitations. A recently published review evaluates measures of and diagnosis of IAH in more detail.^5^

## The reversibility of IAH

In the 1990s, several studies demonstrated that strict avoidance of hypoglycaemia can restore symptomatic awareness of hypoglycaemia in people with IAH.^23–26^ Fanelli et al.^25^ showed that after 3 months of meticulous avoidance of hypoglycaemia, the autonomic symptom response to experimental hypoglycaemia increased, hormonal counter-regulation, including adrenaline, was restored and glycaemic thresholds for triggering these responses partially normalised. Improvement of responses was maintained at 1 year. However, the glucagon response only increased by a magnitude of around 20%, and restoration of symptoms or counter-regulatory responses in those with diabetes duration over 15 years was minimal. Similarly, Cranston et al.^26^ showed that a diligent hypoglycaemia avoidance programme, with a minimum of 3 weeks without a single episode of blood glucose <3.5 mmol/L, improved both symptom response and adrenaline counter-regulation to hypoglycaemia. By contrast, Dagogo-Jack et al.^24^ only found improvement of symptom response, whilst the adrenaline response remained impaired, despite scrupulous avoidance of hypoglycaemia for 3 months. The presence of symptoms might be explained by an increase in beta-adrenergic sensitivity without an increase in peak adrenaline response,^27^ or due to improvements in the responsiveness of the sympathetic nervous system without improvements in the secretory capacity of the adrenal medulla.^28,29^

**Figure 2.**
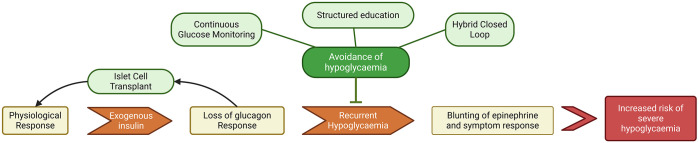
Flow chart of the development of impaired awareness of hypoglycaemia leading to an increased risk of severe hypoglycaemia, and how different interventions aim to disrupt the process to restore awareness. Source: Created with biorender.com.

The strict hypoglycaemia avoidance regimens and intensive treatment programmes underpinning these original studies, some requiring daily clinical visits, would not be feasible within modern clinical practice. Based on the concept that IAH is reversible, multiple studies have sought to identify alternative interventions to restore awareness of hypoglycaemia (Figure 2).^30^ These interventions broadly fall into three categories: technological, educational and pharmacological. Structured educational interventions such as in the ‘Recovery of Hypoglycaemia Awareness in Long-Standing Type 1 Diabetes: Comparing Insulin Pump with Multiple Daily Injections and Continuous with Conventional Glucose Self-monitoring’ (HypoCOMPaSS) study have showed that less stringent avoidance of hypoglycaemia over a prolonged period of time can achieve similar results in terms of symptom restoration and normalisation of the glycaemic thresholds required to initiate counter-regulatory responses.^31^ The most significant improvement in awareness, as determined by the Clarke score, has been demonstrated by islet cell transplantation, with the added benefit of partial restoration of the glucagon response.^32^ However, islet transplantation is expensive, limited as a clinical treatment by cadaveric supply issues and tissue matching, requires life-long immunosuppressant therapy and has a finite time before failure, so in essence, remains for most people, only an effective rescue therapy.^33^ Pharmacological interventions, including beta blockers, diazoxide, naloxone and selective serotonin re-uptake inhibitors, have been trialled in small studies, but currently, no large clinical studies substantiate their routine use in IAH.^34–36^ High intensity interval training has also been proposed as a potential treatment.^37^ Comprehensive reviews of these treatments are outside the scope of this article. The evidence base behind technological interventions will now be evaluated in detail.

## Continuous glucose monitoring

CGM has been a stepwise change in diabetes care in the last two decades. CGM systems sample interstitial fluid and provide a continuous measurement of glucose concentrations at 1- to 5-min intervals, which correlate with blood glucose levels.^38^ CGM systems vary in their capabilities.^39^ Intermittently scanned CGM (isCGM), otherwise known as flash CGM, is reliant upon active scanning of a sensor by a reading device. Real-time CGM (rtCGM) continuously provides up-to-date glucose readings via Bluetooth connection. Glucose alarms have been developed over time. Initially, threshold alarms were introduced, alerting people when their interstitial glucose level crossed a chosen/predetermined threshold.^38^ More recently, ‘predictive’ alarms have emerged, which instead alarm when the blood glucose is predicted to rise above or fall below a certain level. Both types of CGM provide pwT1D and their clinicians with a wealth of data, giving insights on glucose levels and trends, which can be interpreted in a clinical context to inform changes aimed at reducing glucose fluctuations and mean blood glucose levels.^38^

The end result of IAH is an increased risk of SH.^9^ Multiple randomised controlled trials (RCTs) provide evidence that CGM reduces the incidence of SH and improves other hypoglycaemia-related CGM metrics such as time below 3.9 and 3.0 mmol/L.^40^ The HypoDE study was a 6-month multi-centre, open-label, RCT conducted at 12 practices in Germany in pwT1D and either IAH or an episode of SH in the last year, comparing rtCGM to self-monitoring of blood glucose (SMBG).^41^ The incidence risk ratio of SH with rtCGM compared to SMBG was 0.36 (*p* = 0.0247). Similar results were shown in the IN-CONTROL crossover RCT of 52 participants with T1D and IAH, in which there was reduced time spent in hypoglycaemic range (6.8% vs 11.4%) and a significant reduction in episodes of SH with CGM compared to SMBG (14 vs 34 events, *p* = 0.033).^42^ The I HART CGM study showed that rtCGM had a further beneficial impact on hypoglycaemia compared to isCGM.^43^ Thirty-six adults with T1D and IAH or recurrent SH (average Gold score of 5) had a significant reduction in percentage time spent below 3.3 mmol/L using rtCGM versus isCGM (median between-group difference −4.3%, *p* = 0.006). Importantly, these reductions in hypoglycaemia were not associated with significant deterioration in HbA1c.

Real-world data from prospective and retrospective observational studies substantiate the evidence from RCTs.^44–46^ The RESCUE study, a 24-month prospective study of 4441 adults with T1DM in Belgium, showed that people with IAH had the largest reduction in hypoglycaemia from CGM use – there was a reduction from 862 events in the year before CGM initiation, to 119 events/100 patient-years in the year after (*p* < 0.0001).^44^ In the FUTURE study, there was a reduction in the incidence of SH in people with IAH from 36.4% of participants in the last 6 months before CGM to 16% in the 6 months after, even when CGM without an alarm function was used.^47^ In qualitative studies, CGM was seen as helpful by users of the technology in gaining insights into glucose variability, giving an improved sense of control, reducing distress and improving independence from assistance.^48^ However, despite the use of technology, data from an observational study from the T1D Exchange Registry in the United States, including 2074 pwT1D, revealed that the proportion of CGM users reporting more than one episode of SH in the last year was as high as 10.8% (95% confidence interval (CI): 9.1, 12.6).^49^ This indicates that CGM does not abolish SH.

A key question is whether CGM can restore symptomatic awareness through reducing hypoglycaemia exposure. The Freestyle Libre Audit was a retrospective review of 3291 people who commenced on isCGM. The prevalence of IAH, as determined by Gold score, reduced from 28.1% at baseline to 18.1% at follow-up.^50^ Those who restored awareness were more likely to have a shorter duration of diabetes, in concordance with interventional studies in the 1990s.^25^ Ortiz-Zúñiga et al.^51^ presented a prospective observational cohort, initiating isCGM in 60 pwT1D and frequent hypoglycaemia. There was a reduction in time below range (TBR), and also a 47% reduction in the prevalence of IAH, as assessed by Clarke score. As discussed earlier in this review, both the Clarke and Gold scores have limitations, with the Clarke score particularly susceptible to showing improvement in IAH when interventions reduce the frequency of SH, even if symptomatic awareness has not improved.^21^ Furthermore, differentiating between technological and symptomatic awareness is complex. The question in the Gold score ‘Do you know when hypos are commencing?’ can be difficult to answer for pwT1D when their first warning of a hypoglycaemic episode may now be a CGM alarm, as opposed to autonomic symptom recognition.^5^

A number of clamp studies have also sought to answer the question of whether CGM improves awareness. Ly et al.^52^ compared six participants with T1D who had received rtCGM versus five participants who had received standard care. All participants were told to strictly avoid hypoglycaemia. Those using rtCGM had significantly increased adrenaline and symptom responses to experimental hypoglycaemia in comparison with the control group. However, these findings may not be generalisable as all participants were adolescents with a short duration of T1D, whereas the prevalence of IAH typically increases with increased duration of diabetes.^53^ Rickels et al.^54^ aimed to determine whether implementation of rtCGM improves glucose counter-regulation in people with long-standing T1D and IAH. Eleven pwT1D with a mean diabetes duration of 31 years underwent serial hyperinsulinaemic–hypoglycaemic clamps before and after initiation of CGM. The Clarke score and HYPO score, a measure of hypoglycaemia frequency and severity, both significantly improved (*p* < 0.01) without compromise in HbA1c. In response to induced hypoglycaemia, endogenous glucose production, a composite measure of the counter-regulatory response, did not improve by 6 months but significantly improved by 18 months (*p* < 0.05), albeit less than in controls without diabetes. A major limitation of these clamp studies is the small number of participants recruited. T1D Exchange Registry data show that the prevalence of IAH is 31.1% (95% CI: 29.0, 33.2) despite CGM use, supporting the notion that awareness of hypoglycaemia is not fully restored by real-world use of CGM.^49^ Furthermore, Lin et al.^55^ showed that IAH remained a risk factor for SH even with the use of CGM. IAH, as defined by a Gold score ⩾4, was associated with a persistent six-fold increased risk of SH compared to those with NAH despite widespread technology use.

A potential alternative approach to diagnosing IAH is to utilise CGM metrics, as it is proposed that individuals with IAH may exhibit a distinct CGM profile. However, the evidence is mixed. Thomas et al.^56^ reported a small study of 22 pwT1D who wore a blinded CGM and subsequently underwent a hyperinsulinaemic–hypoglycaemic clamp. They demonstrated that a greater CGM TBR was associated with a reduced adrenaline response to hypoglycaemia. Linear regression analysis showed that individually, both TBR and Clarke scores correlated strongly with decreased adrenaline response to hypoglycaemia (*r*^2^ = 0.314 and *r*^2^ = 0.385, respectively) but that there was a weak correlation between them (*r*^2^ = 0.090). It is important to note that the population in this study had higher TBR than usually seen in clinical practice, with a third of the participants exhibiting TBR >19%. Other observational cross-sectional studies have shown correlations between CGM TBR and Clarke scores.^57,58^ By contrast, Choudhary et al.^59^ did not find an association between CGM TBR and Gold scores. In a recent study by Flatt et al.,^60^ CGM metrics such as %Time <3 mmol/L, %Time <3.3 mmol/L and %Time <4 mmol/L were found to be able to predict an absent autonomic symptom recognition to clamp-induced hypoglycaemia. In a post hoc analysis of the ABCD Freestyle Libre audit, including paired TBR, SH and Gold score data for 5029 participants, TBR was found to be significantly associated with both IAH and SH. However, when a %Time <3.9 mmol/L cut-off of 3.35% was used, the AUC for predicting IAH was 0.597, showing poor discriminative ability.^61^ By contrast, the negative predictive value was high at 85%. From these studies, the evidence behind using TBR as a diagnostic tool for IAH is mixed. It appears that a low %TBR can be used to rule out IAH, that much higher levels of %TBR are better associated with IAH, but that in between TBR does not discriminate between normal and impaired awareness. This is an area that requires further research with larger numbers of participants.

In summary, there is clear evidence that CGM reduces the hypoglycaemia burden and incidence of SH in pwT1D and IAH, while not entirely eliminating it. There is some evidence, with significant caveats, that CGM use may restore awareness of hypoglycaemia as determined through questionnaires, but it is unclear if this is true symptomatic awareness or solely technological awareness via alarms. Larger studies using hyperinsulinaemic-hypoglycaemic clamps are needed to definitively answer this question.

## AID systems

AID systems aim to improve glycaemic control, reduce hypoglycaemia and relieve some of the burden of T1D management. The term AID represents a diverse range of devices with many different features.^18^ Hybrid closed-loop (HCL) systems are now the predominant type of AID, and feature automated basal insulin delivery but still require self-inputted meal-time boluses. Older systems, such as sensor-augmented pumps (SAP) or predictive low glucose suspend (PLGS) systems, were able to pause insulin delivery for up to 2 h when sensor glucose fell or was predicted to fall below a preset threshold but were not capable of increasing basal insulin delivery when sensor glucose levels were high.^62^

RCTs have demonstrated improved glycaemic control in pwT1D using AID.^63^ These findings have been confirmed through real-world use of HCL systems in the NHS England pilot,^64^ with an HbA1c reduction of 1.7% and an increase in time in range from 34.2% to 61.9%. From a low baseline (mean 0.37%), no reduction in TBR was demonstrated. Further studies have investigated whether AIDs are beneficial in reducing hypoglycaemia in pwT1D who are prone to hypoglycaemia from prior SH, high burden of hypoglycaemia or presence of IAH. The SMILE study, an open-label RCT in 153 participants with hypoglycaemia-prone T1D, compared PLGS to CGM and continuous subcutaneous insulin infusion (CSII; or standard pump therapy) alone. There were significantly fewer SH events experienced by participants using PLGS (3 vs 18, *p* = 0.0036).^65^ The iDCL Trial Research Group compared HCL with CGM and CSII alone in 72 pwT1D and IAH or an episode of SH in the last 6 months. There was a reduction in TBR by 3.7% (95% CI −4.8, −2.6; *p* < 0.001) with HCL compared to controls.^66^ During the 12-week HCL extension phase, the effects were sustained in the HCL group and replicated in the control group. Further studies support the finding that HCL reduces time in the hypoglycaemic range compared to SAP.^67,68^

Thus, there is reasonably strong evidence that HCL and AID systems reduce the hypoglycaemic burden overall, including episodes of SH in pwT1D and problematic hypoglycaemia. Importantly, both the RCT and real-world data demonstrate that although SH is markedly decreased with AID use, it is not completely eliminated. In an observational survey-based study using the T1D exchange registry, 16.6% of AID users reported an episode of SH in the past 12 months.^49^

An important question is whether AID systems can restore hypoglycaemia awareness and thus reduce the number of pwT1D at risk of SH. A systematic review including four prospective observational studies (*n* = 583) and three RCTs (*n* = 55)^69^ addressed this question. In the observational studies, there was a statistically significant improvement in the Clarke score of 0.45, but this effect size does not meet the minimum clinically significant 1 point difference. In the RCTs, there was no statistically significant effect. The systematic review was limited by the small number of included studies and combined participant count. Several RCTs have shown that HCL or SAP can improve awareness of hypoglycaemia.^62,70–72^ In 95 pwT1D, with a mean age of 18.7 years, Ly et al.^62^ found significant improvements in questionnaire-defined hypoglycaemia awareness using SAP. On the contrary, in a sub-study using hyperinsulinaemic–hypoglycaemic clamps, there was no significant improvement in adrenaline response to hypoglycaemia compared to those using CSII alone.^62^ A prospective study in Spain of pwT1D showed that 6 months after starting HCL, the Clarke score improved from 3.6 to 1.9 (*p* < 0.001) and there was an absolute reduction in prevalence of IAH from 27% to 7%,^72^ albeit with a non-standard definition (Clarke score ⩾3). In 10 participants starting on AID, Malone et al.^71^ reported an improvement in Clarke score from 5.38 at baseline to 3.38 at 18 months. These studies are limited by small numbers, by use of questionnaires susceptible to improvements in frequency of SH and in some, a lack of comparator groups. In real-world data from the T1D exchange registry, regardless of CGM and AID usage, one-third of respondents still reported IAH in the past 12 months.^49^

Three further studies have examined the restoration of awareness after starting HCL with hyperinsulinaemic–hypoglycaemic clamp studies. In a randomised crossover pilot study of 17 participants with T1D and IAH, both adrenergic (5 (4.5–9) vs 4 (4–5.5)) and neuroglycopenic symptom scores (8.5 (6–16) vs 6.5 (6–7)) were higher during hyperinsulinaemic–hypoglycaemic clamps following 8 weeks of HCL than after 8 weeks of standard CSII. This correlated with a significant reduction in the Gold score with HCL (4) compared to CSII (5.5). However, peak adrenaline levels following induced hypoglycaemia were similar after each intervention. The glucose target set during these clamps (2.8 mmol/L) was higher than those used in other clamp studies^22^; this could explain the lack of adrenaline response. Another explanation could be that the adrenaline counter-regulatory response requires a longer duration of HCL therapy to be restored than the symptom response. Lee et al.^73^ compared 26 weeks of HCL use to standard therapy in nine participants. They found no significant change in IAH, assessed through both Clarke and Gold scores. Neither adrenaline response nor autonomic symptom scores significantly increased during experimental hypoglycaemia.

In another study by Flatt et al.,^74^ 10 participants with T1D and IAH (mean age 49 years, mean diabetes duration 34 years) were initiated on HCL (Minimed 670G or t:slim X2).^74^ Hyperinsulinaemic–hypoglycaemic clamps were performed at baseline, 6 and 18 months. The median Clarke score fell below 4 at 12 and 18 months, but the HYPO severity score improved more markedly. Notably, five participants showed little improvement in the Clarke score, and, when experience of SH was removed, the improvement in the Clarke score was not statistically significant. The adrenaline response to hypoglycaemia significantly improved compared to baseline by 6 months, but there was further improvement by 18 months. Autonomic symptom scores only improved at 18 months. These results were additive to those already achieved by rtCGM. Importantly, the results showed a strong negative linear relationship between diabetes duration and improvement in adrenaline response, in line with the earlier work by Fanelli et al.^25^ This raises the question of whether avoidance of hypoglycaemia alone can restore awareness in people with a long duration of T1D. In a post hoc analysis of the WISDM study, evaluating CGM as a treatment to reduce hypoglycaemia, a shorter duration of diabetes was a significant predictor of successful restoration of awareness.^75^ However, in those who improved awareness, the median duration of diabetes was 34.1 ± 4.1 years, indicating that it is possible for people with a long duration of diabetes to restore awareness, albeit with a lower chance of success.

Another important consideration is whether human factors contribute to problematic hypoglycaemia.^76^ In pwT1D who continue to experience SH despite rtCGM, unhelpful health beliefs such as prioritising hyperglycaemia avoidance have been identified. Other health beliefs, such as normalising asymptomatic hypoglycaemia and minimising hypoglycaemia concerns, have also been associated with an increased risk of SH by driving self-management decisions, including excess or early insulin bolusing and insulin stacking.^77^ Flatt et al.^74^ identified a trend towards an increase in the percentage of insulin delivered as bolus in people with IAH, most likely a result of these behaviours, which is at least partially offset by the HCL algorithm. These unhelpful behaviours have been targeted by psychoeducational courses to restore awareness of hypoglycaemia.^78,79^ In the HypoCOMPaSS study, a brief educational intervention was demonstrated to reduce SH rates at comparable levels to CGM or pump, or a combination of both, in pwT1D with IAH.^78^ Some people with T1D and IAH continue to experience problematic hypoglycaemia despite structured education training such as DAFNE (‘Dose Adjustment For Normal Eating’).^80^ Here, psychoeducational programmes such as DAFNE-HART (‘DAFNE-Hypoglycaemia Restoration Awareness training’) and HARP-DOC may significantly reduce SH.^79,81^ To fully exploit the benefits of technology, education for pwT1D on self-management is essential. Furthermore, there may be different phenotypes present within the IAH diagnosis, reflecting differences between individuals in the pathophysiology within IAH.^82^ Certain phenotypes may respond better to specific treatments, dependent upon factors such as comorbid autonomic neuropathy, residual insulin secretion and different patterns of learned behaviours. Further research in studies with larger numbers of pwT1D of a longer duration is needed to explore these factors and determine whether HCL alone can restore awareness in all people with long-standing diabetes and IAH.

## Conclusion

IAH is a common and disabling complication of insulin-treated diabetes, which predisposes people to a significantly increased risk of SH compared to people with NAH. In some individuals, there is reasonably strong evidence that it is a reversible and dynamic condition. There is substantial evidence that technological interventions such as CGM and AID are beneficial in people with IAH, by reducing the frequency of hypoglycaemia, and particularly, SH. The risk of SH in IAH compared to NAH persists despite the use of technology; the ultimate goal should be the restoration of awareness to eliminate this imbalance. It is still unclear whether these interventions consistently restore symptomatic awareness of hypoglycaemia, or whether the effects seen in studies are solely due to technological awareness through alarms, and larger studies are required to test this hypothesis. It may be that a longer duration of hypoglycaemia avoidance is needed to reverse IAH compared with the strict avoidance regimes present in the original reversibility studies. Alternatively, there may be subsets of pwT1D and IAH, for example, those with a longer duration of diabetes, that may not restore awareness with hypoglycaemia avoidance alone. It is not yet possible to answer these questions conclusively due to limitations in the current evidence base, so these areas require further research.

## Appendix

### Abbreviations

AID automated insulin delivery

CGM continuous glucose monitoring

CSII continuous subcutaneous insulin infusion

HCL hybrid closed loop

IAH impaired awareness of hypoglycaemia

isCGM intermittently scanned CGM

NAH normal awareness of hypoglycaemia

PLGS predictive low glucose suspend

PwT1D people with type 1 diabetes

rtCGM real-time CGM

RCT randomised controlled trial

SAP sensor-augmented pump

SMBG self-monitoring of blood glucose

SH severe hypoglycaemia

TBR time below range

T1D type 1 diabetes

T2D type 2 diabetes

## Declarations

**Ethics approval and consent to participate:** This is a review paper. There is no original research with human participants in this article, and informed consent is not required.

**Consent for publication:** Not applicable.

**Availability of data and materials:** Data sharing is not applicable to this article as no datasets were generated or analysed during the review.
